# Protective effect of the daming capsule on impaired baroreflexes in STZ-induced diabetic rats with hyperlipoidemia

**DOI:** 10.1186/1472-6882-10-80

**Published:** 2010-12-22

**Authors:** Jing Ai, Li-Hong Wang, Rong Zhang, Guo-Fen Qiao, Ning Wang, Li-Hua Sun, Guan-Yi Lu, Chao Sun, Bao-Feng Yang

**Affiliations:** 1 Department of Pharmacology, and the State-Province Key Laboratory of Biomedicine and Pharmaceutics, Harbin Medical University, Harbin 150081, P.R. China; 2 Endocrinology and Metabolism Department of the Second Affiliated Hospital, Harbin Medical University, Harbin 150086, P.R. China

## Abstract

**Background:**

The Daming capsule (DMC) is a traditional Chinese medicine used to treat hyperlipoidemia. Both clinic trials and studies on animal models have demonstrated that DMC is beneficial against diabetic symptoms. Impairment of the baroreflex can cause life-threatening arrhythmias and sudden cardiac death in patients with diabetes mellitus (DM). This study was designed to elucidate the effects of DMC on baroreflexes in streptozocin (STZ)-induced diabetic rats with hyperlipoidemia.

**Methods:**

Wistar rats were randomly divided into three groups: untreated controls, rats pretreated STZ and high lipids (a diabetes model or DM rats), and DM rats treated with DMC. The baroreflex sensitivity was examined during intravenous injection of phenylephrine (PE) or sodium nitroprusside (SNP) and quantified by the change in heart rate over the change in mean arterial blood pressure (ΔHR/ΔMABP). Morphological remodeling of baroreceptors was analyzed by transmission electron microscopy (TEM). The mRNA levels and expression of GluR2 and a GABA_A _receptor subunit were measured by quantitative RT-PCR and Western blotting.

**Results:**

Compared to untreated DM rats, DMC significantly elevated the ratio of ΔHR/ΔMABP by enhancing the compensatory reduction in HR (-ΔHR) in response to PE-induced hypertension (+ΔMABP) (*P *< 0.05). In the presence of SNP, DMC increased the ΔMABP (*P *< 0.05). In addition, DMC markedly shortened the duration of blood pressure changes elicited by PE or SNP in DM rats compared to the untreated DM group (*P *< 0.05). Electron microscopy revealed disrupted myelin sheaths, swollen ER, and lysed mitochondria in the nucleus ambiguous (NAm) DM rats. These signs of neuropathology were largely prevented by treatment with DMC for 30 days. Treatment with DMC elevated both mRNA and protein level of GluR2 in the NAm of DM rats, but had no effect on GABA_A _receptor expression.

**Conclusion:**

The Daming capsule partially reversed the parasympathetic baroreflex impairment observed in STZ-induced diabetic rats with hyperlipoidemia. Treatment with DMC also prevented the degeneration of neurons and myelinated axons in the brain stem NAm and reversed the down-regulation of GluR2 mRNA. Rescue of NAm function may contribute to the medicinal properties of DMC in diabetic rats.

## Background

Autonomic neuropathic dysfunctions are common complications in type 2 diabetic patients [1,2]. Impairment of baroreflexes that control heart rate (HR) under conditions of changing arterial blood pressure may contribute to life-threatening arrhythmias and sudden cardiac death [3,4]. Drugs with protective effects on autonomic neuropathy may reduce the mortality induced by diabetes mellitus (DM). Thiazolidinediones (TDs) are hypoglycemic agents that are widely accepted as best treatment for the clinical management of type 2 diabetes. However, inconsistent outcomes limited the usage of TDs for diabetic patients with cardiovascular disease [5,6]. Recently, statins were shown to reduce diabetic nephropathy [7], while a meta-analysis of results from 18,000 diabetic patients across 14 trials revealed that diabetics at "sufficiently high risk" for vascular events could benefit from statins [8-10]. These results suggest that patients with cardiac dysfunction could profit from lipid-modulating drugs. Nevertheless, long-term treatment with statins could lead to rhabdomyolysis. Therefore, it is essential to develop alternative drugs to prevent the blunted baroreflex sensitivity induced by DM.

The Daming Capsule (DMC) is a combination of traditional Chinese herbs with a variety of medicinal properties. Our previous study indicated that DMC therapy significantly reduced total cholesterol (TC) and low density lipoprotein (LDL) content in hyperlipoidemia patients with few adverse side effects [11]. Further study demonstrated that DMC could reverse the prolonged QT and PR interval and improve cardiac function in diabetic rats. This cardioprotective effect may be mediated, at least partially, by modulating the mRNA transcription and protein expression of Kv4.2 and α_1c _and by preventing DM-induced dysfunction of cardiac myocytes [12].

It is well known that cardiac function is regulated by baroreflex circuitry and that baroreflex sensitivity is usually impaired in patients with DM [1]. Given the broad spectrum of potential DMC benefits, in particular the robust cardioprotective responses, we investigated the effects of DMC on baroreflex sensitivity in diabetic rats with hyperlipoidemia.

## Methods

### Preparation of DMC

The present formulation of the DMC was designed by the Department of Pharmacology of Harbin Medical University and prepared by Harbin Yida Ltd. The preparation of DMC was described in detail in our previous publications [11,12]. Briefly, *Rheum palmatum *(Solanaceae) was harvested from Gansu province, China, after 3 years of cultivation. *Cassia obtusifolia *L. (pulse family) and Panax ginseng C.A. (*Acanthopanax gracilistylus*) were harvested from Sichuan province after a 1-year cultivation period, and *Salvia miltiorrhiza *(Labiatae) was obtained from Jilin province after an 8-year cultivation period. These plant materials were all collected from September to November. Voucher specimens of *Rheum palmatum *(1.008.1), *Cassia obtusifolia *L. (6.048.1), Panax ginseng C.A. (1.058.1), and *Salvia miltiorrhiza *(1.080.1) were deposited at the Heilongjiang Food and Drug Administration. The samples were ground into powder and cased in capsules in a *Rheum palmatum*: *Cassia obtusifolia *L.: *Salvia miltiorrhiza*: Panax ginseng C.A. ratio of 12:12:6:1. All processes followed the regulations of the State Food and Drug Administration of China.

### Quality control by quantitative analysis of the total anthraquinones

Quality control standards and quantitative analysis of DMC were described in our previous publication [12]. Briefly, the total anthraquinone content was determined by the marker compound chrysophanol using high performance liquid chromatography (HPLC). Chromatographic separation employed a ZORBAX C18 column (250 mm × 4.6 mm, 5 μm) and a mobile phase composed of methanol-1% phosphate acid solution (85:15). The flow rate was 1.0 ml/min and anthraquinones were detected by 254 nm absorbance. Only DMC samples with more than 1.5 mg of chrysophanol in each 300 mg capsule were used in the present study.

### Animals

Male Wistar rats (200-230 g, *n *= 40) were obtained from the Animal Center of the Second Affiliated Hospital of Harbin Medical University, China, and housed under controlled temperature (23 ± 1°C) and humidity (55 ± 5%) with a 12 h-12 h light-dark cycle. The study received approval from the ethics committee of Harbin Medical University (HMUIRB-2008-06).

### Establishment of diabetic animal model

In accordance with our previous study [12], fat emulsion was prepared with lard (20%), thyreostat (1%), cholesterol (5%), sucrose (5%), fructose (5%), sodium glutamate (1%) and salt (6%), and emulsified in 20% Tween 80 with 30% propylene glycol. The emulsion was stored at 4°C until use. All rats were equally and randomly divided into 3 groups. The control rats (Ctr) group received 0.9% NaCl by oral administration. The diabetic model group (DM) first received fat emulsion (10 ml/kg) by oral administration for the first 10 days and then were given streptozocin (STZ, in 0.1 M citrate buffer solution, pH 4.2, Sigma) at 40 mg/kg by intraperitoneal (i.p.) injection once daily for 3 d. Fasting blood glucose (FBG) levels were measured 72 h after the last STZ administration using a Grace glucometer (Grace Medical, Inc. America) to ensure induction of diabetic symptoms. Rats with FBG levels higher than 16.7 mmol/l or 300 mg/dl were judged to successfully model DM in rats. These rats were then treated with the fat emulsion (10 ml/kg) for 30 d. The FBG was tested during this period to monitor diabetic status. The DMC-treated rats (both controls and DM model rats fed the fat emulsion) were given 100 mg/kg/d DMC for 30 d.

### Biochemical Estimations

Blood was collected from heart, then separated and analyzed for total cholesterol (TC) and triglycerides (TG) using appropriate kits (Nanjing Jiancheng Bioengineering Institute, China).

### Surgical procedure

Rats were anesthetized with sodium pentobarbital (40 mg/kg) by i.p. injection. Supplemental doses of anesthetic (0.1 ml of 1% sodium pentobarbital) was administered every 30 min to maintain deep sedation as indicated by lack of eye blink and withdrawal reflexes. For blood pressure measurements, the ends of short polyethylene-50 tubes were tapered down to 0.5 mm to form the insertion ends of catheters. The left femoral artery and right femoral vein were exposed and the tapered tips of the two catheters (both filled with heparinized saline) were inserted. Vasoactive drugs were injected into the femoral vein through one catheter and blood pressure was monitored through the femoral artery by connecting the catheter to a blood pressure transducer (MlT0699, AD instruments). The transducer was positioned at heart level.

### Baroreflex sensitivity

Arterial blood pressure (ABP) was measured using the BL-420 Data Acquisition & Analysis System (Chengdu Tme Technology Co., Ltd. China). Mean arterial blood pressure (MABP) and HR were then recorded automatically. A series of phenylephrine (PE) injects at 16, 32, 64, 128, and 256 μg/ml was used to elevate BP. Alternatively, sodium nitroprosside (SNP) at 10, 20, 40, 80, then 160 μg/ml was used to lower BP. Both drugs were injected in a fixed volume (0.04 ml/100 mg). The next drug concentration was injected only when the HR and ABP level had reached a plateau from the preceding injection. The ΔHR response (maximal HR - baseline HR) to the ΔMABP (maximal MABP - baseline MABP) induced by injections of PE or SNP were used to construct dose-response and baroreflex sensitivity curves. For dose-response curves, ΔHR/ΔMABP was plotted as the function of PE and SNP dose. The ΔHR to ΔMABP relationship were also plotted to illustrate the ΔHR changes induced by ΔMABP. All curves were fitted to the Boltzmann equation using Prism 5.0 software [13-19].

### Transmission electron microscopy (TEM) Analysis

The location of the nucleus ambiguous (NAm) was identified as previously described [20]. Tissue samples were prepared by routine methods for TEM analysis. In brief, 1.0 mm Epon embedded sections through the NAm were stained with uranyl acetate and lead citrate and examined by JEOL 1200 electron microscope (JEOL Co., Japan).

### Quantitative RT-PCR analysis

Total RNA was prepared from rat brain stem (±600 μm from 0 point), and mRNA levels of alpha-amino-3-hydroxy-5-methyl-4-isoxazole propionate (AMPA) receptor subunit 2 (GluR2: F: TGTCCTCCTTTCTCCCT, R: CTGAACCATCCCTACCC) and gamma aminobutyric acid A receptor (GABA_A_)(F: CTGAAGTGAAGACGGACAT, R: ACGCAGGAGTTTATTGG) were measured using the SYBR Green PCR Master Mix Kit (Ambion, Austin, TX, USA) on a 7500 FAST Real-Time PCR System (Applied Biosystems, Foster City, CA, USA) with the U6 gene as an internal control (F: GCTTCGGCAGCACATATACTAAAAT, R:CGCTTCACGAATTTGCGTGTCAT).

### Western blot analysis

The expression of proteins in brain stem tissues (±600 *μm *from 0 point) was detected by Western blot analysis. The proteins examined were GluR2 (88 kDa) and GABA_A _(70 kDa). The primary antibodies and HRP-conjugated secondary antibodies were all purchased from Santa Cruz Biotechnology (Santa Cruz, CA, USA).

Tissues were lysed in 600 μl lysis buffer containing 1% protease inhibitor solution, and then centrifuged at 12,000 × g for 30 min to collect the soluble protein extracts from the supernatant. The protein concentration was determined by a Sunrise-Basic Tecan microplate reader (Tecan, Austria) using bovine serum albumin as the standard. Protein samples were resolved on 10% SDS-PAGE gels and transferred to PVDF membranes (Bio-Rad, Hercules, CA, USA). The PVDF membranes were then incubated with the indicated primary antibodies diluted at 1:3,000 in PBS buffer for 1 h at room temperature. Inhibitory peptides were used in some trials to confirm antibody specificity. After washing three times in PBS-T (10 min each wash), the PVDF membranes were incubated for 1 h at room temperature with HRP-conjugated secondary antibodies (1:5,000) in PBS-T blocking buffer containing 5% dry milk. Protein bands were then detected with the Odyssey infrared imaging system (LI-COR, Lincolin, NB, USA). An antibody to β-actin at 1:10,000 was used as the loading control. The SDS-PAGE gels were stained with Coomassie blue solution before the electrotransfer of proteins to verify the quantity of the protein recovered and the quality of separation. Densitometry analysis was performed with Quantity One software (Area × OD), and the density of each protein was normalized to β-actin. The normalized expression levels of target proteins are presented as fold changes over the expression of the control samples.

### Statistical analysis

Data are presented as means ± S.E.M. Statistical comparison of baseline MABP and HR between groups was determined using Student's t-tests. To compare the dose-response curves between treatment groups, one-way ANOVA with repeated measures were used. *P *< 0.05 was considered statistical significant. All curves were plotted using Graphpad Prism 5.0 software.

## Results

### Biochemical Estimations

**Figure 1:**
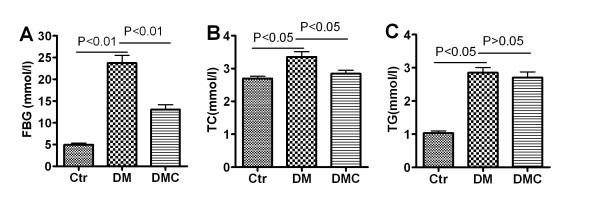
**Effects of DMC on FBG, TG and TC in diabetic rats**. FBG: fasting blood glucose; TG: triglycerides; TC: total cholesterol. *n *= 10.

In rats administered with STZ and then fed the high-fat emulsion for 30 days, fasting blood glucose (FBG), triglycerides (TG), and total cholesterol (TC) were all significantly elevated (Figure 1), indicating that diabetic and hyperlipoidemia symptoms were successfully established in the DM group. Daily treatment with DMC for 30d dramatically decreased both FBG and TC levels in the second cohort of these lipid-fed DM rats (DM + DMC). In contrast, no significant change in TG was observed in DMC-treated diabetic rats compared to DM rats (Figure 1C).

### Effects of DMC on resting blood pressure and heart rate in anaesthetized rats

**Figure 2:**
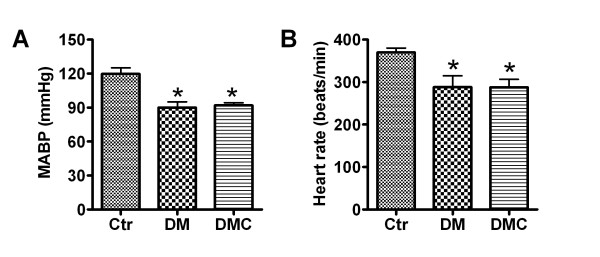
**Resting mean arterial blood pressure (MABP) and heart rate (HR) in control, DM model rats, and DM rats treated with DMC**. Data are presented as Mean ± S.E.M. * mean value was significantly different from that of the control group (*P *< 0.05). *n *= 10.

After rats were anaesthetized by sodium pentobarbital, resting MABP and HR were measured before PE or SNP application. In control rats, MABP and HR were 119.78 ± 2.6 mmHg and 369.83 ± 5.1 beats/min and DMC had no effect on either basal MABP or HR in control rats (data not shown). In DM rats, MABP and HR were reduced to 90.05 ± 2.5 mmHg and 288.67 ± 12.9 beats/min. Treatment with DMC did not significantly alter baseline MABP and HR in DM rats (*P *> 0.05; Figure 2A and 2B).

### Baroreflex control of HR during PE application

**Figure 3:**
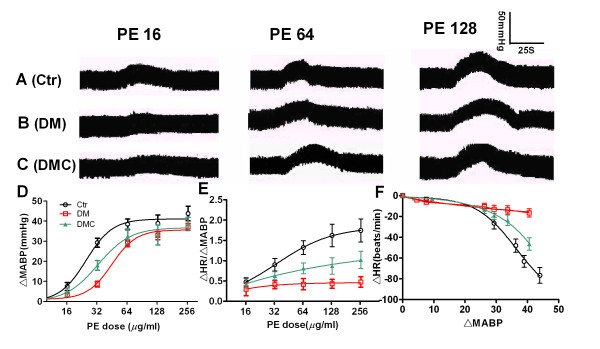
**Effect of DMC on the attenuation of baroreflex sensitivity in DM rats following PE administration**. A: Normal rat (Ctr). B: DM rat (DM). C: DMC-treated DM model rat (DMC). Trace: blood pressure changes induced by PE application at 16, 64, and 128 μg/ml. D) Dose dependent ΔMABP curve of rats; E) Dose dependent Δ HR/Δ MABP curve of rats; F) Correlation of Δ HR to Δ MABP in rats at different PE dosages. *n *= 8.

Gradual increases in MABP were measured in all experimental rats following intravenous injections of PE (Figure 3A, B and 3C). Compared with the untreated control group, the PE-induced ΔMABP increase was significantly suppressed in DM rats as indicated by the rightward shift in the PE-ΔMABP curve (Figure 3D, P < 0.05). When DM rats were treated with DMC for 30 d, however, the PE-ΔMABP curve was shifted leftward toward the control curve (*P *< 0.05). The ratio of ΔHR/ΔMABP is also an index of parasympathetic baroreflex sensitivity and was significantly decreased in DM rats compared to untreated control rats. The ratios were enhanced after DMC administration, indicating partial rescue of the baroreceptor reflex. For example, at a PE dose of 128 μg/ml, ΔHR/ΔMABP was 1.74 ± 0.28 in control rats, 0.47 ± 0.12 in DM rats, and 1.02 ± 0.16 in DM+DMC rats (Figure 3E, P < 0.05). Parasympathetic baroreflex sensitivity is also described by the compensatory change in HR (ΔHR) as a function of the change in blood pressure (ΔMABP) during physiological or pharmacological perturbations (e.g. PE injections) [19]. Figure 3F illustrates that DMC significantly increased the -ΔHR response in PE-injected DM rats. Indeed, the relation between ΔHR to ΔMABP was virtually flat in DM rats, indicating near total suppression of the baroreceptor reflex. In the DM+DMC group, however, a ΔMABP greater than 30-40 mmHg was associated with a decrease in heart rate, indicating partial rescue of the reflex. No effect of DMC on the ΔHR to ΔMABP of untreated control rats was observed (data not shown, *P *> 0.05).

### Baroreflex control of HR during SNP application

**Figure 4:**
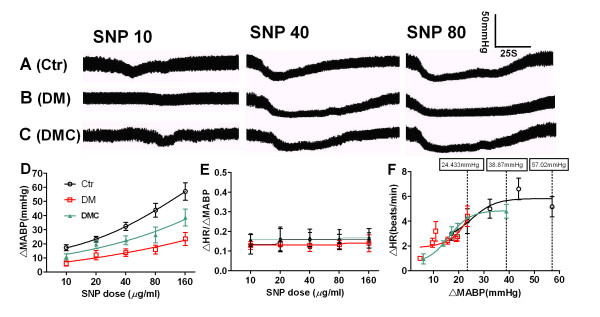
**Effect of DMC on the attenuation of baroreflex sensitivity in DM rats following SNP administration**. A: Normal rat(Ctr). B: DM rat(DM). C: DMC-treated DM rat (DMC). Trace: blood pressure changes induced by SNP application at 10, 40, and 80 μg/ml. D) Dose dependent Δ MABP curve of rats; E) Dose dependent Δ HR/Δ MABP curve of rats; F) Correlation of Δ HR to Δ MABP in rats at different PE doses. *n *= 8.

The baroreflex response mediates both a decrease in HR during rising MABP and an increase in HR in response to reduced MABP (Figure 4A, B and 4C). Decreases in ΔMABP induced by different SNP dosages were significantly attenuated in DM rats as indicated by the rightward shift in the SNP-ΔMABP curve. Again, the SNP-ΔMABP relation was partially reversed by DMC treatment (*P <*0.05). The SNP dose-dependent curves for sympathetic baroreflex sensitivity (ΔHR/ΔMABP versus SNP) and ΔHR versus ΔMABP were plotted (Figure 4E, F) but showed no differences between control, DM, and DM + DMC groups (Figure 4E, *P *> 0.05). However, DMC treatment did markedly elevate the SNP-induced ΔMABP level in DM rats (Figure 4D, F and Table 1). For example, at 160 μg/ml, the maximum ΔMABP was 57.02 mmHg in control rats, 24.33 mmHg in DM rats and 38.87 mmHg in DM +DMC rats. The sympathetic baroreflex sensitivity was not changed in control rats after administrated with DMC (data not shown, *P *> 0.05)

**Table 1:** Arterial blood pressure and HR before and after PE and/or SNP administration

		Baseline	PE (256 μg/ml)	SNP(160 μg/ml)	HR range
Ctr	MABP(mmHg)	119.78 ± 2.61	162.78 ± 3.64*	62.76 ± 3.24*	
	HR (mmHg)	369.83 ± 5.04	293.13 ± 5.52*	374.99 ± 6.21	81.86 ± 4.27
DM	MABP (mmHg)	90.05 ± 2.47^#^	127.17 ± 4.25*	66.62 ± 4.56*	
	HR (beats/min)	288.67 ± 8.84^#^	272.17 ± 6.26	293.07 ± 7.36	20.90 ± 4.05
DMC	MABP (mmHg)	91.93 ± 1.14^#^	130.61 ± 4.25*	53.4 ± 4.62*	
	HR (mmHg)	287.5 ± 6.31^#^	241.2 ± 5.36*	292.29 ± 3.47	51.09 ± 5.17

Ctr: control group; DM: diabetic mellitus; DMC: Daming capsule; MABP: Mean arterial blood pressure; HR: heart rate; PE: phenylephrine; SNP: sodium nitroprusside. Data are presented as Mean ± S.E.M. ^# ^Mean value was significantly different from that of the control group (*P *< 0.05). *Mean value was significantly different from that of the baseline (*P *< 0.05). *n *= 8.

### DMC shortened the ABP recovery time after PE or SNP application in DM rats

**Figure 5:**
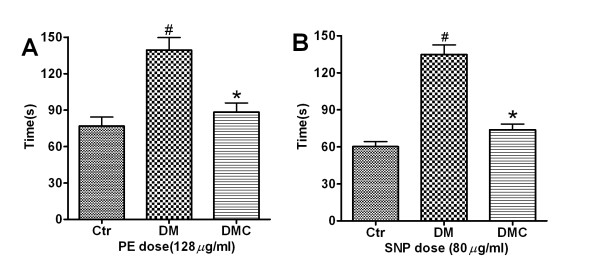
**DMC significantly shorten the prolonged recovery time of ABP changes induced by PE or SNP in DM rats**. A) Durations of ABP increasing level after PE (128 μg/ml) application; B) Durations of the decreased ABP level after SNP (80 μg/ml) application. Data are presented as Mean ± S.E.M. *n *= 6.

The recovery time of arterial BP changes following PE or SNP injection is another key index used to evaluate baroreflex sensitivity [21]. Our results demonstrated that the time of recovery to baseline after drug treatment was significantly longer in DM rats compared to untreated control, while DMC significant reduced the (elongated) duration of the PE-induced and SNP-induced ΔABP. For example, at a PE dose of 128 μg/ml, the duration to recovery from peak ABP to baseline was 76.83 ± 18.2 s in control rats, 139.3 ± 25.3 s in DM rats, and 88.2 ± 18.8 s in DM + DMC rats (Figure 5A, P < 0.05). Similarly, when the SNP dose was 80 μg/ml, the durations to recovery were 60.2 ± 10.1 s, 134.8 ± 19.3 s, and 73.83 ± 11.6 s (Figure 5B, P < 0.05). Thus, DMC did improve the biphasic baroreceptor response, at least using drug sensitivity and recovery time as indices.

### DMC reversed the ultrastructure remodeled in NA of DM rats

**Figure 6:**
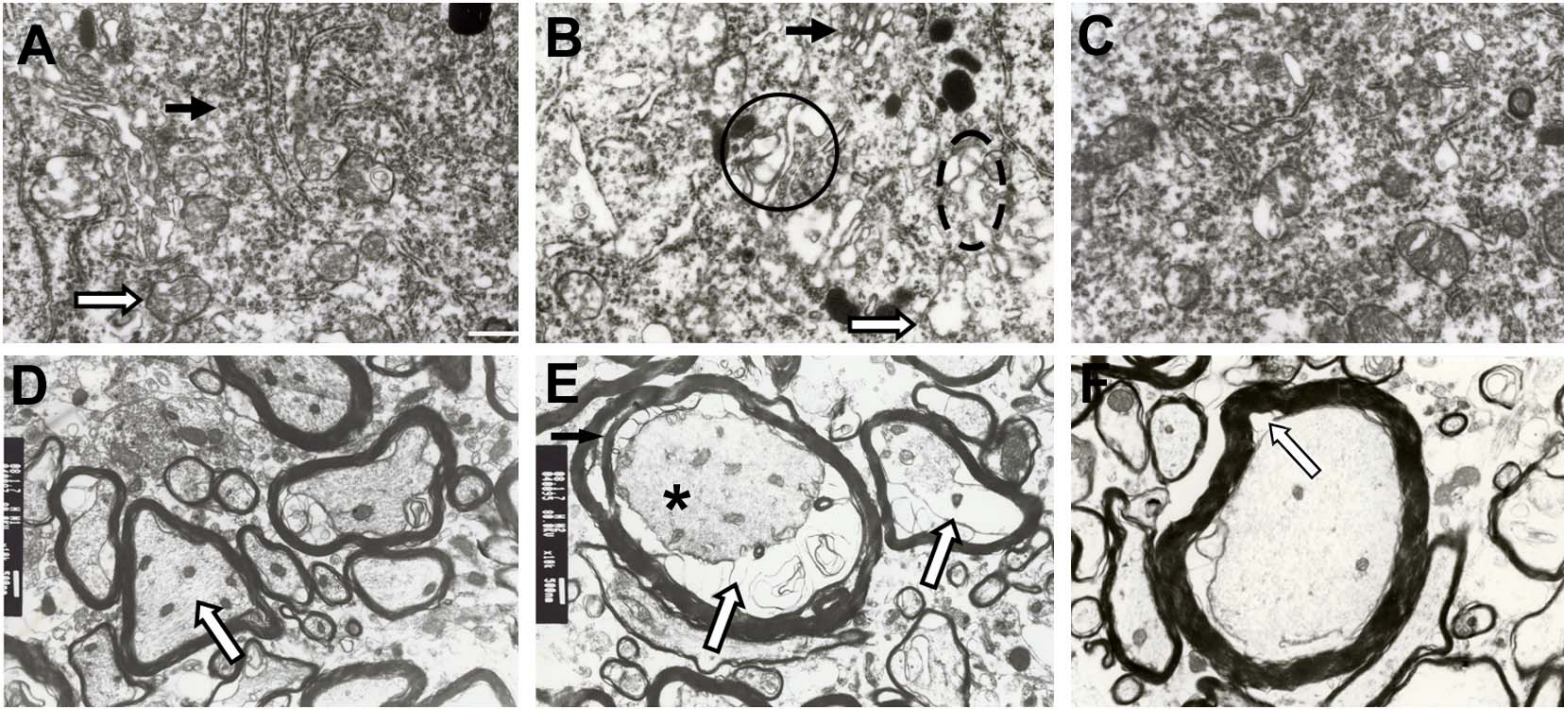
**Electron micrographs showing the ultrastructural characteristics of NAm neurons in normal, DM, and DMC-treated DM rats**. A: Electron micrograph of cytoplasm from a NAm neuron in a normal rat. The mitochondria (indicated by a hollow arrow) and endoplasmic reticulum (indicated by a solid arrow) are the predominant cytoplasmic organelles. B: Electron micrograph of the cytoplasm from a NAm neuron in a DM rat. The expanded endoplasmic reticulum is circled by a dashed line ellipse, the expanded Golgi body is circled by a solid line, and the partially lysed mitochondrion is indicated by a hollow arrow. C: Electron micrograph of the cytoplasm from a NAm neuron from a DMC-treated DM rat showing regular mitochondrial and endoplasmic reticulum structure. D: Electron micrograph of myelinated nerves from the NAm in a normal rat. Microtubules, microfilaments, and neurofilaments are visible in the medullary sheath and the membranes of myelinated nerves are attached tightly to the inner wall of the medullary sheath. E: Electron micrograph of myelinated nerves from the NAm in a DM rat. Axons appear to detach from the myelin sheath, yielding enlarged paces between the axolemma and medullary sheaths (hollow arrows). Decreased electron density indicates partial dissolution of the axoplasm (* symbol). F: Electron micrograph of myelinated nerves from the NAm a DMC-treated DM rat showing a reduction in the spaces between the axolemma and medullary sheaths (indicated by hollow arrow). Scale bar: 1.0 μm.

Transmission electron micrographs revealed swelling of the endoplasmic reticulum and mitochondria, as well as damage to the Golgi body complex in NAm neurons of DM rats (Figure 6A and 6B). Again, treatment with DMC markedly reversed these signs of neuropathology (Figure 6C). In DM rats, the myelin surrounding NAm axons was often damaged and axons were of smaller diameter from cytoplasmic shrinkage, leading to voids between the myelin and axolemma (Figure 6E). These morphological signs of nerve damage, however, were not observed in DMC-treated rats (Figure 4F).

### DMC significantly elevated mRNA and protein expression of GluR2 of DM rats

**Figure 7:**
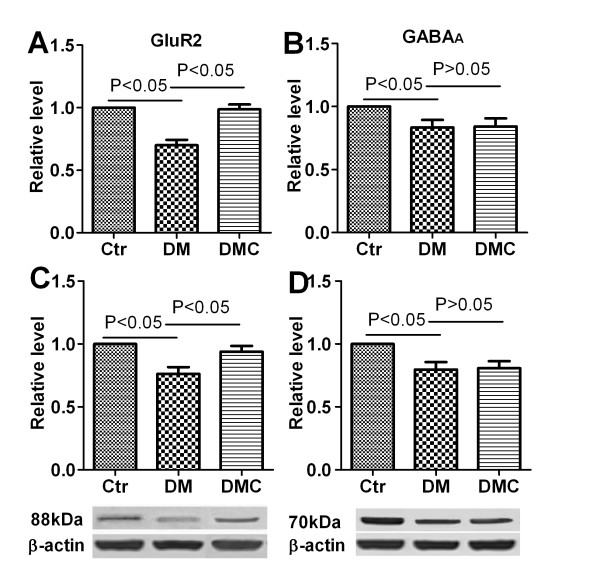
**mRNA and protein expression levels of GluR2 and GABA_A _receptor**. The relative mRNA levels are shown in A (GluR2) and B (GABA_A_) and the relative protein levels are shown in C (GluR2) and D (GABA_A_). Data are presented as Mean ± S.E.M. *n *= 6.

Finally, we examined the expression of AMPA-type GluR2 and GABA_A _receptors, as these are the major excitatory and inhibitor transmitters that mediate signal transduction between baroreceptors and the brain stem NAm. The DM rats demonstrated significant down regulation of GluR2 and GABAA receptor subunit mRNA and protein (Figure 7). Both the down-regulation in GluR2 mRNA and protein were reversed by DMC treatment in DM rats (Figure 7A and 7C, *P *< 0.05). In contrast, DMC did not restore mRNA levels or expression of GABA_A _receptors in DM rats (Figure 7B and 7D, *P *> 0.05).

## Discussion

The Daming pill (DMC) was designed from several Chinese herbs and possesses a multitude of medicinal effects. Indeed, our previous study demonstrated that DMC was cardioprotective in DM rats by improving both diastolic and systolic functions of the heart and by protecting individual cardiac myocytes against DM-associated morphological rearrangements [12]. The present study underscores and extends the cardioprotective potential of DMC by demonstrating that DMC treatment also improved the baroreflex sensitivity in DM rats by increasing the sensitivity of the compensatory ΔHR response during pharmacologically-induced ΔMABP. In addition, DMC reduced the time required for recovery from ΔMABP perturbations induced by PE and SNP. Further evidence suggested that DMC preserved the functional integrity of baroreceptor neurons by preventing neuronal damage in the nucleus ambiguous and by restoring the expression of AMPA receptors containing GluR2.

In the present study, we found that long-term DMC treatment significantly improved the impaired parasympathetic baroreflex gain in DM rats as indicated by both the ΔHR versus ΔMABP relation and the time to recovery following PE treatment. In DM rats, this relation was flat (no ΔHR in response to ΔMABP), indicating that the baroreceptor reflex was severely compromised in DM rats. In contrast, no improvement in baroreceptor sensitivity was found in response to SNP application as measured by the ΔHR versus ΔMABP relation. However, DM rats already had reduced MABP and HR, so the dynamic range may have been reduced below the threshold range of the rat baroreceptors. Indeed, heart rate did not change with blood pressure changes below 30 mmHg even in healthy untreated rats. Under low blood pressure, a compensatory increase in heart rate may not have been efficiently signaled through the baroreceptor reflex. In order to further assess the actions of DMC, the recovery time following PE and SNP treatment were assessed. The results clearly demonstrated that the prolonged recovery time in DM rats treated with SNP was improved by DMC treatment, indicating that DMC could improve some aspects of BP regulation even when BP was reduced.

Baroreceptor sensitivity is exquisitely sensitive to cellular energy charge [22] and we observed damaged mitochondria, in addition to dilated endoplasmic reticula in NAm neurons of DM rats. The Daming capsule effectively prevented these signs of neuropathology in DM rats and hence preserved the function of the brain stem. Under normal conditions, myelination improves axonal conduction velocity by restricting current flow to nodes and decreasing membrane capacitance [23]. Abnormal myelination, including myelin sheath atrophy or thickening, can reduce impulses propagation velocity or cause conduction failure. The result that DMC improved the impaired baroreflex sensitivity may be explained, at least in part, by prevention of the DM-associated atrophy of NAm neurons and axons that mediate the brain stem baroreceptor reflex. Both glutamate and GABA also play important roles in maintaining baroreflex sensitivity. The release of glutamate could decrease blood pressure through the action of glutamate on AMPA receptors containing GluR2 [24,25] while GABA increases blood pressure by inhibiting the parasympathetic system circuit via GABA_A _receptors [26,27]. Our findings suggested that the protective effects of DMC on baroreflex control of heart rate could be due to the upregulation of GluR2 and a concomitant increase in synaptic transmission or neuronal excitability within the baroreceptor reflex pathway.

## Conclusion

In summary, DMC prevented the parasympathetic baroreflex impairment in the STZ-induced diabetic rat model with hyperlipoidemia. The mechanisms may be related to the elevated sensitivity of the baroreflex conferred by improved neuronal function in the NAm.

## Competing interests

The authors declare that they have no competing interests.

## Authors' contributions

JA designed and carried out the studies, performed the statistical analysis, and drafted the manuscript. LHW carried out the studies and performed the statistical analysis. RZ and LHS performed the investigation of baroreflex sensitivity. GFQ and NW, GYL carried out the qRT-PCR and Western blot investigations. BFY supervised, designed and drafted the manuscript. All authors read and approved the final manuscript.

## Pre-publication history

The pre-publication history for this paper can be accessed here:

http://www.biomedcentral.com/1472-6882/10/80/prepub
